# Generative models of MRI-derived neuroimaging features and associated dataset of 18,000 samples

**DOI:** 10.1038/s41597-024-04157-4

**Published:** 2024-12-05

**Authors:** Sai Spandana Chintapalli, Rongguang Wang, Zhijian Yang, Vasiliki Tassopoulou, Fanyang Yu, Vishnu Bashyam, Guray Erus, Pratik Chaudhari, Haochang Shou, Christos Davatzikos

**Affiliations:** 1 Center for AI and Data Science for Integrated Diagnostics (AI2D), Perelman School of Medicine, University of Pennsylvania, Philadelphia, PA 19104 USA; 2 Artificial Intelligence in Biomedical Imaging Laboratory (AIBIL), Perelman School of Medicine, University of Pennsylvania, Philadelphia, PA 19104 USA; 3 Department of Radiology, Perelman School of Medicine, University of Pennsylvania, Philadelphia, PA 19104 USA; 4 Department of Electrical and Systems Engineering, University of Pennsylvania, Philadelphia, PA 19104 USA; 5 Department of Biostatistics, Epidemiology and Informatics, Perelman School of Medicine, University of Pennsylvania, Philadelphia, PA 19104 USA

**Keywords:** Biomarkers, Neuroscience, Machine learning

## Abstract

Availability of large and diverse medical datasets is often challenged by privacy and data sharing restrictions. Successful application of machine learning techniques for disease diagnosis, prognosis, and precision medicine, requires large amounts of data for model building and optimization. To help overcome such limitations in the context of brain MRI, we present GenMIND: a collection of generative models of normative regional volumetric features derived from structural brain imaging. GenMIND models are trained on real brain imaging regional volumetric measures from the iSTAGING consortium, which encompasses over 40,000 MRI scans across 13 studies, incorporating covariates such as age, sex, and race. Leveraging GenMIND, we produce and offer 18,000 synthetic samples spanning the adult lifespan (ages 22-90 years), alongside the model’s capability to generate unlimited data. Experimental results indicate that samples generated from GenMIND align well with the distributions observed in real data. Most importantly, the generated normative data significantly enhances the accuracy of downstream machine learning models on tasks such as disease classification. Dataset and the generative models are publicly available.

## Background & Summary

Empowered by widely available open datasets and challenges, in the past decade, machine learning algorithms have surpassed human-level performance on many non-trivial computer vision and natural language understanding tasks including face recognition, object detection, question answering, and translation^1–4^. Translating the success of artificial intelligence tools from natural image or language applications to medical domain holds immense potential for advancing automated diagnosis and precision medicine. However, medical data usually contains sensitive patient information, which is subject to stringent privacy regulations from hospitals^5,6^. Consequently, machine learning research in medicine is contained within individual institutes or hospitals, constrained by relatively small sample sizes due to resource limitations and resulting in datasets that lack diversity in terms of patient demographics and pathology representation. Models trained on such less-representative medical data are subject to sample selection bias and may lack generalizability across clinical cohorts^7–10^. This underscores the need for collaborative efforts to access larger and more diverse medical datasets, ensuring the development of robust and trustworthy AI models in healthcare. To address such data shortages, specifically in the context of neuroimaging, numerous international consortia have been established. Examples include ENIGMA^11^ and ISTAGING^12^, which primarily compile structural, functional imaging, and genetic data from tens of thousands of individuals. However, the usage of the data is still restricted to each consortium’s participants. To broaden accessibility and enhance research potential, generative models in machine learning have garnered attention. Techniques such as Kernel-Density Estimation (KDE)^13^ and generative adversarial networks (GANs)^14–16^ have shown promise in synthesizing data that faithfully represents the underlying probability distributions of real-world data. This capability opens avenues for generating synthetic datasets that closely resemble actual patient data, thereby augmenting the pool available for analysis and model training. Recently, these models have been utilized to produce synthetic medical data and have proven valuable in many medical applications including chest X-ray screening and skin lesion detection^17–20^. These synthetic datasets can be openly shared and distributed without concerns regarding data privacy. Furthermore, access to synthetic data that accurately represents a large population can significantly benefit downstream classification models, especially when labeled samples are scarce. By leveraging synthetic data, we can bridge the gap between the available labeled samples and the diverse real-world scenarios, improving the robustness and generalization of our models.

In many studies involving MRI (Magnetic Resonance Imaging), brain structure is commonly summarized by region-of-interest (ROI) volumes^21^, which are derived from structural T1-weighted MRI scans. ROI volumes are robust brain features that have been validated in many applications including disease diagnosis and prognosis^22–26^, progression modelling^27–31^, and pathology subtype discovery^32–35^.

In this paper, we present GenMIND, a collection of generative models that generate normative ROI data over the adult lifespan (age range: 22 to 90 years) for different demographic groups categorized by race and sex. Furthermore, using these models, we offer a dataset of 18,000 synthetic neuroimaging samples representing a diverse global healthy adult population. Notably, we also provide the generative models alongside the dataset to enable users to customize their data synthesis. The dataset includes participants’ demographic information, such as sex, age and race, which can be beneficial for research focusing on mitigating algorithmic bias and promoting fairness. Our approach to generating synthetic data involves training generative models, specifically KDE models, on 34,000 subjects from the iSTAGING consortium^12^ to synthesize both brain anatomical ROI volumes and their associated demographics. In the experiments, we assess the quality of the synthetic data from both qualitative and quantitative perspectives. For example, we create plots highlighting the similarity between the real and synthetic data distributions across all ages groups. In terms of quantitative evaluation, we use statistical tests and machine learning models to show the similarity in distributions between synthetic and real data. We also examine the fidelity of the synthetic data by performing covariates prediction including sex or race classification and age regression tasks. Finally, we show the utility of the synthetic data in real world applications such as brain age gap prediction^36^.

GenMIND, a comprehensive structural brain imaging feature generator and dataset, aims to significantly contribute to advancing machine learning research within the field of neuroimaging. In particular, GenMIND facilitates: 1) Local Disease Population Comparisons: Researchers can leverage GenMIND to compare their own patient populations to GenMIND’s normative dataset. To ensure robust inference that is invariant to inter-site differences, researchers should first use part of their control data to harmonize their measures with those of GenMIND. 2) Brain Age Prediction Models: GenMIND serves as a resource for training brain age prediction models. These models are used to detect the effects of neurodegenerative and neuropsychiatric disorders on the brain^36^. In the technical validation section, experiments using GenMIND explore the relationship between brain age residuals and cognitive scores. Beyond brain age prediction, the synthetic data generated by GenMIND can also be leveraged to train other machine learning models and adapt them for smaller-scale studies through techniques like transfer learning or domain adaptation. 3) Enriching Healthy Controls in Classification Studies: The dataset enhances the healthy control class for discriminative analysis and disease subtyping research. For instance, in an Alzheimer’s disease diagnosis experiment, GenMIND is utilized for data augmentation, leading to more robust results. 4) Synthetic Data Generator Models: Beyond providing the GenMIND dataset containing 18,000 samples, researchers also have access to the synthetic data generator models. These pre-trained models allow customization of brain volume synthesis based on factors such as sex, age range, and race groups.

Our goal is to continue expanding GenMIND with additional covariates, including genetic risk factors, cognitive scores, and biomarker data. We also aim to build synthetic data generation models for other imaging modalities, such as DTI and fMRI. This expansion will enable users to enrich their own datasets by synthesizing highly specific brain measures tailored to their individual studies.

## Methods

**Fig. 1:**
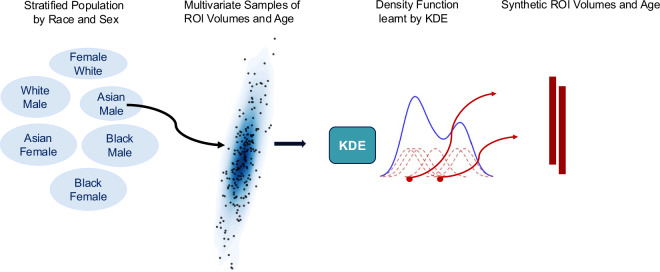
Work flow of **GenMIND**: KDE-Based Distribution Learning of Volumetric ROIs and Age Stratified by Race and Sex. This flowchart outlines our approach for learning the multivariate distribution of 145 volumetric ROI measures and age across different race and sex categories. First, we stratify the population into subsets using race and sex (e.g., white females, black males, etc.). For each category, we assemble multivariate samples of ROI volumes and age from the real dataset. KDE is then used to learn the density function specific to each category. This approach enables category-specific distribution learning and supports balanced data generation for downstream tasks.

In the following subsections we describe i) the real dataset used to construct the GenMIND generative model, and ii) the approach used for training the generative model for synthetic data generation. Figure 1 illustrates the workflow of the synthetic data generation process.

### Real data

For our real data, we use the iSTAGING consortium^12^ that consolidated and harmonized imaging and clinical data from multiple cohorts spanning a wide age range (22 to 90 years). Our data consists of multimodal neuroimaging and demographic measures taken from subjects labeled as cognitively normal in the iSTAGING consortium. Specifically, the neuroimaging measures are the 145 anatomical brain ROI volumes (119 ROIs in gray matter, 20 ROIs in white matter and 6 ROIs in ventricles) from baseline scans extracted using a multi-atlas label fusion method^21^. To mitigate site effects, ComBat-GAM harmonization^37^ was applied to these 145 ROI volumes while accounting for age, sex and intracranial volume (ICV). The demographic measures including subjects’ age, sex, and race were accounted in the synthesis.

To address the race and sex imbalance in the iSTAGING dataset and prevent it from affecting synthetic data generation, we use a stratified approach. Subjects in the real data are stratified based on race and sex covariates, resulting in six subsets or categories: white male, white female, black male, black female, asian male, and asian female. Before fitting the generative model, within each category, the feature vector of 145 ROI volumes along with the age variable is normalized using the mean and the standard deviation. Mean and standard deviation values for each category are retained to facilitate the back transformation of synthesized data from normalized space to the original volume space.

### Generative model training

We employ a separate non-parametric KDE model for each category, using a Gaussian kernel to delineate the joint probability density of age and the 145 ROI volumes. The selection of the Gaussian kernel is crucial; its smooth, bell-shaped profile is well-suited for generating a continuous representation of the underlying distribution from discrete observations.

Within this framework, each data point is represented by a local Gaussian density surface centered at the observed location. Collectively, the overall density is smoothly estimated using composite functions of these local densities, based on the discrete empirical data. This approach facilitates the estimation of the multivariate joint probability density function for ROI volumes and age within each distinct category. Generating new synthetic data involves a two-step process: model fitting and sampling. Initially, a multivariate KDE model is fitted to the real data of each sex and race combination with carefully tuned bandwidth parameters. Subsequently, we generate new data points by sampling from this refined distribution. Each synthesized vector is of size 146; the first 145 elements correspond to the ROI volumes, and the last element represents age. The sampling process introduces randomness, ensuring that while the synthetic data are not exact replicas of the original, they adhere to the same statistical properties.

### Hyper-parameter selection

The bandwidth parameter determines the width of the Gaussian kernel and plays a crucial role in KDE. A smaller bandwidth results in a more granular estimate with greater detail, reflecting minor variations in the data distribution, whereas a larger bandwidth leads to a smoother and more generalizable estimate. By carefully selecting the bandwidth, the KDE model can effectively navigate the trade-off between overfitting, where the model captures excessive noise, and underfitting, where important features of the data distribution are overlooked. To identify the optimal bandwidth for the kernel in KDE, we conducted a grid search spanning bandwidths from 0.5 to 1. The selection criterion for the optimal bandwidth is the maximization of the log-likelihood score across these values. For the majority of the categories analyzed, the optimal bandwidth converges at approximately 0.7. The search space of the bandwidth is constrained to 0.5 to 1 because our dataset is normalized (~*N*(0, 1)). A small bandwidth overfits the training data, since it fits a narrow Gaussian kernel around any point; whereas a large bandwidth, close to 1, results in an overly smoothed density estimate that fails to maintain the correlation between the 145 ROIs and age. The model implementation and the bandwidth grid-search are carried out using the scikit-learn library^38^.

## Data Records

The GenMIND dataset is available at NITRC^39^. The “genmind_dataset.csv” file contains 18,000 synthetic samples with 3000 samples allocated per combination of race and sex, a deliberate sampling strategy aimed at ensuring thorough representation across demographic groups. Each sample comprises of 148 features (145 brain ROI volumes along with age, sex, and race information). See Table 1 for an example of the synthetic dataset. We enhance the utility of our dataset by providing users with access to both the synthetic data and the fitted KDE models. The KDE models are hosted at https://huggingface.co/spaces/rongguangw/GenMIND. This interface equips researchers with the ability to generate synthetic data that aligns precisely with their research requirements.

**Table 1:** Synthetic data table comprising of brain ROI volumes along with demographic information.

SampleID	Sex	Race	Age	3rd Ventricle	4th Ventricle	Right Accumbens Area	…
Synth1	F	Black	83.49359824	572.7620447	1298.91257	438.5023773	…
Synth2	M	White	62.13155172	1204.073174	1149.880788	246.0560264	…
.	.	.	.	.	.	.	.
.	.	.	.	.	.	.	.
.	.	.	.	.	.	.	.

Sex variable is coded as F for female and M for male.

## Technical Validation

We conduct a comprehensive validation of the synthetic data through two levels of analysis. In the first level, we employ a combination of statistical and machine learning techniques to ensure that the synthetically generated brain ROI volume data closely mirrors the distribution of real ROI volumes across demographic variables such as age, sex, and race. Subsequently, at the second level, we aim to validate the practical use of synthetic data in real-world scientific applications, such as brain age gap estimation and data augmentation. Through these rigorous analyses, we aim to ascertain the accuracy, reliability, and utility of synthetic data in both replicating real data distributions and facilitating meaningful applications in scientific research and clinical practice.

### Assessing Fidelity: Statistical and Machine Learning Analysis Comparing Synthetic and Real Data Distributions

**Fig. 2:**
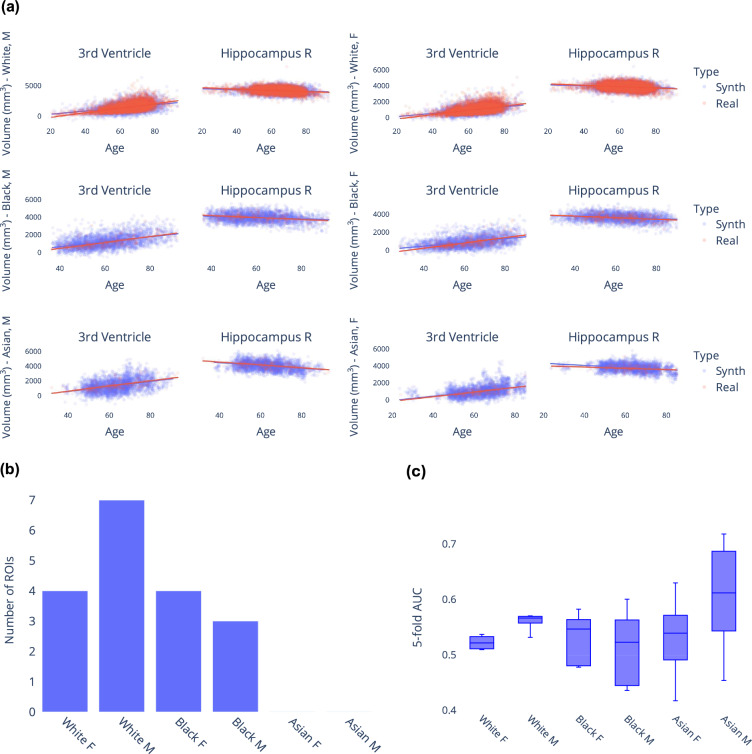
Comparing synthetic and real data distributions. (**a**) Qualitative assessment of real versus synthetic ROI volume data distributions in the 3rd ventricle and right hippocampus. (**b**) Number of ROIs with significant difference in mean volume in each category; results are from univariate statistical analysis that check for difference in synthetic and real ROI volumes. (**c**) 5-fold cross-validated AUC scores for binary classification of real and synthetic data using support vector machine regression indicate that the generated dataset is indistinguishable from the real dataset.

In this section, we examine synthetic samples generated from the KDE models in comparison with a held-out dataset containing real samples from the iSTAGING consortium^12^. Given that the synthetic data spans the entire adult lifespan and covers all combinations of race and sex, Fig. 2a serves to visually illustrate the similarity in distributions between real and synthetic data across several ROIs. From Fig. 2a it is evident that for these two representative examples, i.e. 3rd ventricle and right hippocampus, there is significant overlap between the generated data samples and the real data samples. Furthermore, the age trend observed in the synthetic data roughly mirrors that observed in the real data. To further evaluate the fidelity of the ROI volume distributions, we conduct univariate statistical analysis via linear regression of each ROI volume on group (real versus synthetic) while adjusting for age as a covariate^40^. We apply Bonferroni correction^41^ to adjust for multiple comparisons, resulting in a significance threshold of $\alpha =\frac{0.05}{145}=0.0003$. Figure 2b presents the count of ROIs, out of the total 145 ROIs, with statistically significant differences in group means for every combination of race and sex. While the univariate statistical analysis checks for group average differences, we can also measure individual-level correspondence between the real and synthetic datasets using the simple permutation-based intermodal correspondence (SPICE) test^42^. Using the SPICE test, within each category, we can assess whether age-matched real and synthetic samples have higher similarity in ROI volumes compared to unmatched pairs. Pearson correlation is used as the measure of similarity, and 999 permutations are carried out for the test. For each category, we report the observed correlation (mean  ± standard deviation) of ROI volumes in age-matched pairs along with the p-value of the permutation test: *r* = 0.991 ± 0.0064, p-value = 0 (white females); 0.988 ± 0.0080, p-value = 0 (white males); 0.988 ± 0.0089, p-value = 0.016 (black females); 0.989 ± 0.0069, p-value = 0.007 (black males); 0.990 ± 0.0043, p-value = 0.008 (asian females); and 0.987 ± 0.0066, p-value = 0 (asian males). Using a significance threshold of 0.05, we note that across all categories, we reject the null hypothesis, concluding that the similarity of ROI volumes in age-matched real and synthetic pairs is higher than in unmatched pairs. This demonstrates that there is high correspondence between real and synthetic data.

Additionally, to assess the similarity between the multivariate ROI volume distributions of synthetic and real samples, we employ a support vector machine (SVM)^43^ classifier. The SVM classifier is trained to differentiate between synthetic and real data using ROI volumes as input features. We perform this analysis for each category of race and sex. We use a radial basis function (RBF) kernel in the SVM, as it is effective in capturing non-linear relationships between features. The model is trained using 5-fold cross-validation. The classifier’s performance is evaluated using the area under the receiver operating characteristic curve (AUC)^44^. An AUC of 0.5 would suggest that the SVM is making random predictions and cannot differentiate between real and synthetic samples, indicating that the two datasets are indistinguishable. Figure 2c summarizes the findings across different race and sex categories. While there remains some classification capability (AUC > 0.5 in white and asian males), suggesting slight differences between the two distributions, subsequent analyses under practical applicability section demonstrate that this discrepancy does not confound or critically impact the utility of the synthetic dataset.

**Fig. 3:**
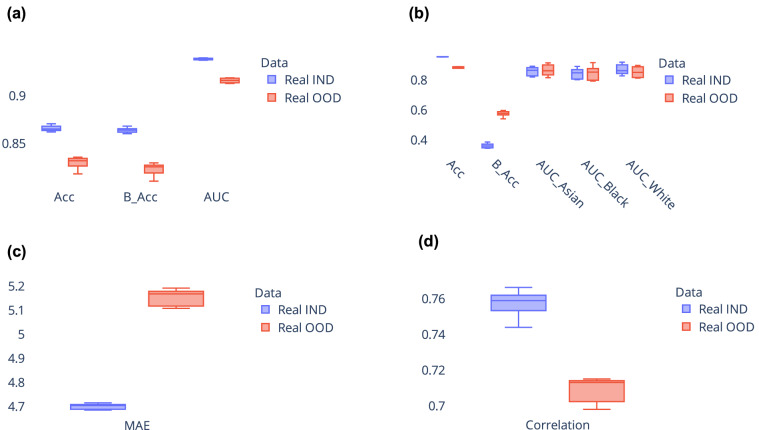
Comparison between model trained on real data and model trained on synthetic data for covariate prediction. Results from (**a**) sex classification, (**b**) race classification, and (**c**), (**d**) age regression. Results are shown from 5-fold cross validation. Real IND refers to results from model trained on real data and tested on held-out real data, Real OOD refers to results from model trained on synthetic data and tested on held-out real data. Acc: Accuracy, B_Acc: Balanced Accuracy, AUC: Area under receiver operating characteristic curve, MAE: Mean absolute error.

Since the data for each combination of race and sex is generated from different KDE models, it is critical to assess whether the relationship between ROI volumes and covariates (age, sex, race) in the synthetic data aligns with observations from real data. To carry out this analysis, we build machine learning models for covariate prediction, specifically using gradient boosted trees (implemented using XGBoost library^45^). These models are trained to predict covariates based on ROI volume data, performing tasks such as age regression, sex classification, and race classification. By comparing the performance of the models trained on synthetic data with those trained on the real data, we determine if synthetic data is a suitable substitute for real data. Sex and race classification performance is evaluated using metrics such as accuracy, balanced accuracy, and AUC. Age regression performance is evaluated using mean absolute error (MAE) and Pearson’s correlation^46^ between predicted age and ground truth age. Figure 3 shows the results for covariate prediction, we used 5-fold cross-validation during analysis. While models trained on real data generally outperform those trained on synthetic data, it is noteworthy that the performance of models trained on synthetic data is comparable to their real data counterparts. These results suggest that synthetic data can serve as a valuable alternative to real data, when the latter is not available. The next section delves into the practical applicability of synthetic data, exploring its potential benefits and limitations in real-world scenarios.

### Practical Applicability

In addition to validating the quality of the generated data, we conduct comprehensive assessments to showcase the practical applications of synthetic data across various scenarios. This includes its efficacy in augmenting training datasets for disease classification and deriving clinically meaningful estimates of brain age gaps. In this section, we utilize the ADNI study as a held-out dataset to assess the aforementioned properties. The synthetic data is derived from a KDE model that was retrained on the remaining studies, following the steps outlined in Methods.

#### Data augmentation

**Fig. 4:**
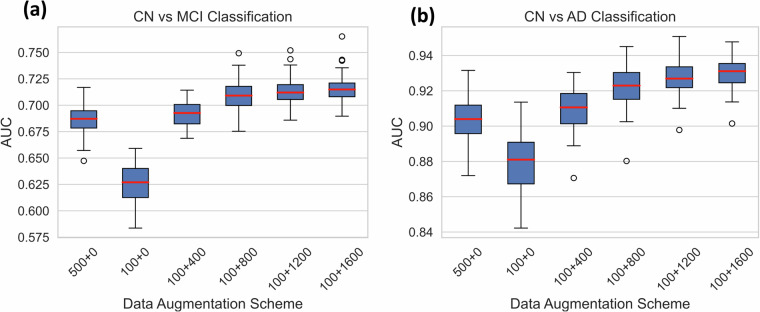
Performance of disease classification models that use synthetic data during training. AUC scores for (**a**) CN vs MCI and (**b**) CN vs AD classifications using different data augmentation schemes. The X-axis lists out the different schemes, for instance 100+400 describes experiment where model is trained using a control data set comprising of 100 real samples and 400 synthetic samples. The classification process is performed 50 times for each augmentation scheme, using fifty random splits of training and testing sets.

Deep learning methods usually require a large number of training samples, which are laborious and costly to obtain, especially for brain MRI studies. Moreover, datasets focusing on specific diseases may sometimes lack a sufficient number of healthy controls. To address this, we investigate the feasibility of using synthetic data to supplement the normal control (CN) group. We center our analysis on the mild cognitive impairment (MCI) or Alzheimer’s disease (AD) classifications. Leveraging data from the ADNI study^47^, we evaluate whether synthetic data based data augmentation can help improve classification performance. We divide the ADNI dataset, allocating 500 cognitively normal participants for training, 368 for testing, and evenly dividing 1101 MCI and 419 AD participants between training and testing sets. To ensure the robustness of our findings, we conduct fifty distinct random splits. For both CN vs MCI and CN vs AD classifications, we train the SVM algorithm on the training set and assess its performance on the test set using the AUC metric. Notably, the training sets comprise different proportions of CN data from both real and synthetic datasets, allowing us to evaluate the specific contribution of synthetic data to performance enhancement (Fig. 4).

Figure 4 illustrates that a reduced number of training samples from the normal control group (500 v.s. 100) results in lower MCI and AD classification performances, underscoring the importance of augmenting the healthy control dataset. Supplementation of the CN set with synthetic normal control data progressively improved classification AUCs, although the rate of improvement slowed as the proportion of synthetic data within the CN set increased.

#### Brain age gap estimation

Brain age gap^36,48–51^, the difference between predicted brain age and actual chronological age, indicates deviations from normal brain aging and proves important for assessing neurological health. Utilizing large-scale synthetic control data can potentially enhance the development of age-prediction models, offering more reliable and clinically relevant brain age gap estimations. We train XGBoost regression models on both synthetic and real control data, and use the trained models for calculating brain age gaps for CN and MCI/AD participants in the held-out ADNI dataset. We refer to brain age gaps derived using the synthetic data model as OOD brain age gaps and refer to brain age gaps derived using the real data model as IND brain age gaps. To mitigate biases inherent in predicted brain age estimates, we apply Cole’s method^52^ before calculating the brain age gaps. Further, to examine their effectiveness in indicating cognitive decline and underlying neuropathology, we examine their correlations with Mini Mental State Examination (MMSE) scores — a widely utilized cognitive assessment for measuring cognitive impairment.

**Fig. 5:**
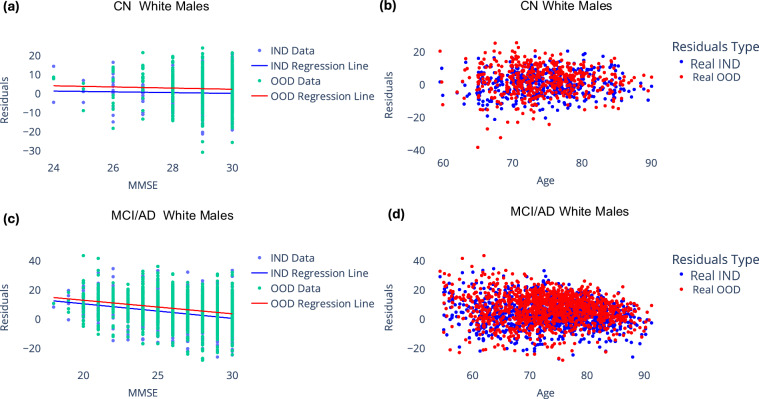
Comparison of brain age gap for ADNI participants estimated using a model trained on real data and another model trained on synthetic data. (**a**) and (**c**) show the bias corrected brain age gap (residuals) versus MMSE scores for subjects labelled as CN and MCI/AD respectively. (**b**) and (**d**) show the bias corrected brain age gap (residuals) versus age for subjects labelled as CN and MCI/AD respectively. Real IND refers to results from model trained on real data and tested on held-out real data, Real OOD refers to results from model trained on synthetic data and tested on held-out real data.

As shown in Fig. 5, both OOD and IND brain age gaps show no significant correlations with MMSE among CN participants. However, among MCI/AD participants, they exhibit significant correlations (*p* < 0.0001). Interestingly, the Pearson’s correlation coefficient for OOD brain age gaps (*ρ* = − 0.235) is similar to that observed for IND brain age gaps(*ρ* = − 0.304). This further underscores the potential of regression models trained on extensive synthetic datasets to provide brain age gap estimation with increased clinical significance.

## Usage Notes

By employing rigorous statistical and machine learning analyses, we demonstrate that the synthetic data generated by GenMIND closely aligns with real data distributions across various demographic variables, including age, sex, and race. Our evaluations, detailed in the technical validation section, indicate that the multivariate distributions learned by the model accurately preserve covariate effects, ensuring that the synthetic data maintains the integrity of the original data’s demographic characteristics. The practical utility of GenMIND has been illustrated through various applications, showing its potential to serve as either a substitute or complement to real data.

### Usage by Researchers and Clinicians

Researchers and clinicians can use GenMIND’s synthetic data in a variety of ways. For instance, in scenarios where real-world datasets suffers from class imbalances (e.g., underrepresented populations), they can expand their sample sizes with synthetic data to improve machine learning model performance and increase the statistical power of their studies. One key application of GenMIND is in training models for brain age prediction, for estimating brain age gap — an established image-derived biomarker to assess an individual’s brain health^48^. Additionally, GenMIND can be used for normative modeling^53^, a statistical framework that is becoming increasingly popular in neuroscience. Normative modeling involves building a reference model that captures the typical variation in brain measures, such as brain volume, within a healthy population. Since GenMIND is designed to emulate a global healthy population across the human lifespan, it is well-suited for constructing these reference models. Researchers can use these models to study how various factors, including neurodevelopmental and neurodegenerative disorders, affect the brain by comparing their cohort’s brain measurements to the reference models. Clinicians, on the other hand, can apply normative modeling in a clinical setting to assess individual patients by identifying deviations from expected brain measures, potentially aiding in diagnosis or tracking disease progression. Moreover, synthetic data holds significant potential for harmonization efforts in integrating and comparing datasets from diverse sources. Synthetic data can be specifically tailored to generate covariate-matched control data, ensuring that external studies can be effectively harmonized with existing datasets. This approach enables researchers to create control groups with similar demographic characteristics as that of their study population, thereby reducing confounding effects and then utilizing a harmonization technique^54^ to align their external data to the synthetic matched control data. This enhances the robustness of comparative analyses.

### Using the Hugging Face Interface

GenMIND generative models are accessible as a web tool on Hugging Face. This platform features a customized data generation tool that allows users to select parameters such as sex and race, along with sample size. Upon submission, the tool generates a downloadable CSV file containing synthetic data sampled from the pretrained generative models for the specified sex and race.

While we demonstrate the potential uses of synthetic data, there are certain limitations that end-users should consider when using GenMIND. The quality of synthetic samples is influenced by the selection of the bandwidth parameter in KDE, and although the synthetic data statistically resembles real data, it may contain noise due to kernel smoothing. This noise might affect the results of any analysis performed with GenMIND, so users should interpret their results with caution. Additionally, when integrating their own data with GenMIND, users should be aware of potential site-related differences that might impact their analyses. There are techniques available that can help the user harmonize their data to GenMIND^55^ to mitigate site-related effects and facilitate more robust analyses. Also, as the sample sizes increase, the uniqueness of synthesized samples decreases. This occurs because the expressiveness of the KDE model is limited by the density function learnt from the finite training data. Essentially, as more samples are drawn from a fixed KDE model, the diversity of the samples decreases, since the KDE does not introduce new variability beyond the learned density. As a result, the samples tend to become more densely packed and similar to each other, reducing their overall uniqueness.

In conclusion, GenMIND represents an advancement in the generation and application of synthetic neuroimaging data, providing a valuable resource for the neuroimaging community. Furthermore, the KDE models used in GenMIND can also be applied to features derived from other modalities, such as functional or diffusion MRI, to learn and replicate feature distributions in these domains. By continuing to expand GenMIND with additional covariates, including genetic risk factors, cognitive scores, and biomarker data, we aim to further enhance its utility and applicability in diverse research contexts. This work underscores the importance of synthetic data in advancing neuroimaging research, promoting data accessibility, and ensuring the development of robust, generalizable machine learning models in healthcare.

## Data Availability

All our model building and analysis were carried out in python. All our data transformations are described in detail in the methods section. Statistical analyses were conducted via online python packages, statsmodels 0.8.0, SciPy 1.6.3, NumPy 1.16.6 and pandas 0.21.0. Our machine learning experiments were conducted with version 1.1.3 of the Scikit-learn library (https://scikit-learn.org). The trained KDE models are available at https://huggingface.co/spaces/rongguangw/GenMIND. Sample code for model training and data generation is available at https://huggingface.co/spaces/rongguangw/GenMIND/blob/main/script/synthetic_data_generation.ipynb.
